# On the Therapeutical Value of the Injection of Medicated Fluids through the Eustachian Tube

**Published:** 1881-05

**Authors:** Jos. Gruber, Lucien Howe


### ON THE THERAPEUTICAL VALUE OF THE INJECTION OF MEDICATED FLUIDS THROUGH THE EUSTACHIAN TUBE.—EXTRACTS FROM A PAPER BY PROF. JOS. GRUBER.

FROM THE GERMAN BY DR. LUCIEN HOWE.

THE subject of this paper was not only deemed worthy of discussion at the last meeting of the British Medical Association, but is a question of such eminent practical value as to be entitled to frequent consideration in the proceedings of medical societies.

I have been, for many years, a zealous advocate of this treatment. Ehrard, also practices the same method, and when W. Kramer, of Berlin, denied even the possibility of injected fluids entering the middle ear, then did Schwartz and Weber-Liel*,

* *Zeitschrift fuer Pract. Heilkunde.* Wien, 1864.

and I furnish direct proofs to the contrary. Each of us arrived at the same conclusion by a different course, and thus refuted some statements* in the report of a committee, in Berlin, which appeared adverse to the method. We showed, that even with the drum of the ear in a normal condition, liquids, injected through the Eustachian tube, not only could, but actually did, reach the middle ear. We would not have discussed the question more at length, had we not also been convinced of the value of such methods of injection. * * * *

After establishing the underlying facts concerning the possibility of this kind of treatment, there arise two questions of special interest, namely:

*a*) Can such fluids, injected through the Eustachian tube, have any particular action?

*b*) In what manner do they produce their effects?

* * * * It would seem that every book on Materia Medica gave an answer to the question, as to whether fluids injected into the middle ear through the Eustachian tube produced any benefit; for, I would ask, why should not an astringent liquid have the same effect applied to the mucous membrane of the middle ear, as it has upon the lining membranes of the posterior nares, or of the bronchi? Why should not a fluid, which would remove the inflammatory products from similar cavities in the body, lined by mucous membrane, exert a like influence in the middle ear? Why should not a fluid, which can produce a healthy reaction, or even an inflammation on other mucous membranes, be able also to effect the middle ear? And why should we not expect that the ear would be acted upon in a special way by any agent, which, on other membranes, produces a result that may be considered specific. I think all these questions, and other similar ones asked by the practical aurist, are answered by clinical experience in a manner favorable to the method under discussion. * * * *

No one pretends that mucous or any other products of inflammation left in the middle ear, can really be "dissolved." A

* *Deutsche Klinik*, No. 12, 1865, and No. 2, 1866.

knowledge of the form and character of such secretions precludes that idea. But it is of great advantage to so alter them by means of fluids injected through the Eustachian tube as to make them more loose and freely movable, so that they may be pushed aside or rendered less obnoxious.

For example, solutions of potassium and sodium really have no power to dissolve the hardened masses of mucous and epithelium, and yet they can cause them to become so loose that they can be extruded from the cavity of the middle ear on inflating it with air, or can be moved off toward the mastoid cells, where they are not as injurious. Indeed, by making an artificial opening in the membrane, they may in the same manner be pushed straight out of the ear, and in some cases, where perforation already exists, no such preparatory operation is necessary. It is true, if the fluid be injected through the tube in an aimless fashion—as is too frequently the case—nothing is gained. But when the accompanying circumstances are taken into consideration, such injections have often been found of use where simple inflation of the ear with air has given little or no satisfaction.*

* * * * As to the choice of the fluid which is to be injected, I think we may follow the same general rules which guide us in the application of substances to the mucous membrane of the pharynx. Even in the cartilaginous portions of the tube, weak solutions of nitrate of silver are very well borne, but, of course, when the stronger are used, care must be taken not to introduce them too high up for fear of producing violent inflammation. In making such applications to the upper portions of the tube, the choice will be regulated somewhat by the end to be attained. For example, solutions of potash or soda are useful in loosing the hardened products of inflammation, and Weber-Liel has lately recommended the carbonate of soda, which seems to be specially well adapted for the purpose. In chronic cases, however, where the membrana tympani is intact,

* Compare Jos. Gruber—On the Accumulation of Inflammatory Products in the Middle Ear.—*Allgemeine Wiener Med. Zeitung*, 1879, No. 27.

I think the favorable result is more frequently due to the mechanical effect of the injection and to the resulting stimulation, than to the medicine itself. * * * * There are two methods of injecting these fluids into the middle ear, when that cavity is closed externally by the membrane which has not been perforated. One of these is the injection through the catheter, which has been passed into the orifice of the tube. The other is the method already described in my text-book, of "introducing medicated fluids into the Eustachian tube without the aid of a catheter," This is practically as follows: A proper quantity of the solution is placed in a small blunt-pointed syringe and injected in a suitable manner through one nostril, while at the same time the opposite nostril is held more or less firmly closed by the other hand of the surgeon. The degree, to which this second nostril is opened or closed, will, of course, regulate the amount of pressure exerted by the fluid, and the distance which the substance enters the tube, or its mechanical effect, can thus be regulated by the amount of pressure used. I acknowledge even more frankly than before, that more unequal results are obtained by this method, than by the use of the catheter; but, only after this has been properly tried, is it possible to determine as to the best form of manipulation in introducing the fluid? I have long contended that the injection made through an Eustachian catheter, was often of no avail, for the substance sometimes touched only a portion of the mucous membrane of the ear. * * * * Indeed, I believe that we have, in the injection of medicated fluids through the Eustachian tube, a most valuable method of treatment in certain forms of ear troubles. In other cases it can be relied on only as an aid in the general plan adopted, but when properly employed, an assistance is thus gained which greatly helps to crown our efforts with success.—*Monatsschrift fuer Ohrenheilkunde*, Jahrgang XIV, No. 9.

